# Effects of Three Types of Oil Dispersants on Biodegradation of Dispersed Crude Oil in Water Surrounding Two Persian Gulf Provinces

**DOI:** 10.1155/2012/981365

**Published:** 2012-01-26

**Authors:** Azadeh Zolfaghari-Baghbaderani, Mozhgan Emtyazjoo, Parinaz Poursafa, Sedigheh Mehrabian, Samira Bijani, Daryoush Farkhani, Parisa Mirmoghtadaee

**Affiliations:** ^1^Islamic Azad University North Tehran Branch, Tehran, Iran; ^2^Iranian Offshore Oil Company, Tehran, Iran; ^3^Faculty of Marine Science and Technology, Islamic Azad University North Tehran Branch, Tehran, Iran; ^4^Environment Research Center, Isfahan University of Medical Sciences, Isfahan, Iran; ^5^Department of Biology, Faculty of Science, Teacher Training University, Tehran, Iran; ^6^Oil Industry Research Center, Tehran, Iran; ^7^Department of Clinical Pharmacy and Pharmacy Practice, School of Pharmacy, Isfahan University of Medical Sciences, Isfahan, Iran

## Abstract

*Objective*. To determine the most effective and biodegradable dispersant of spilled oil in water surrounding two Persian Gulf provinces. *Methods*. This study compared the effects of three dispersants, Pars 1, Pars 2, and Gamlen OD4000 on removal of oil in two Persian Gulf provinces' water. Overall, 16 stations were selected. Using the Well method, the growth rate of isolated bacteria and fungi was identified. To specify the growth rate of microorganisms and their usage of oil in the presence of the above-mentioned dispersants, as exclusive sources of carbon, the bacteria were grown in culture medium for 28 days at 120 rpm, 30°C, and their optical density was measured by spectrophotometry. Then, we tested biological oxygen demand (BOD) and chemical oxygen demand (COD) in microorganisms. *Results*. The highest growth rate was documented for the growth of microorganisms on either Pars 1 or Pars 2 dispersants or their mixtures with oil. However, the culture having microorganisms grown on Pars 1 had higher BOD and COD than the other two dispersants (9200 and 16800 versus 500 and 960, *P* < 0.05). Mixture of oil and Pars 2 as well as oil and Pars 1 dispersants showed the highest BODs and CODs, respectively. In the Bahregan province, microorganisms grown on Pars 2 had maximum amount of BOD and COD in comparison with Pars 1 and Gamlen dispersants (7100 and 15200 versus 6000 and 10560, *P* < 0.05). *Conclusion*. Pars 1 and Pars 2 were the most effective dispersants with highest degradability comparing Gamlen. In each region, the most suitable compound for removing oil spill from offshores with least secondary contamination should be investigated.

## 1. Introduction

The main causes of oil pollution in the oceans are extraction of oil, transportation with ballast water release and tanker accidents, and also war-related incidents [1]. Unfortunately, such incidents are not uncommon. Land and offshore oil wells also can be a source of oil spills into ocean water. Oil spills from such accidents may quickly spread over many square miles of water surface. The spills are particularly destructive for local wildlife and plant life when they get close to shorelines. They also damage boats, fishing gear, and harbor installations and greatly diminish the value of the shore as recreational resources [2–4].

The use of dispersants in nearshore areas is expected to increase the exposure of aquatic organisms to petroleum [5]. If not treated, crude oil spills would require long period of time to naturally biodegrade; it nearly takes 22 years for complete biodegradation of one kilogram of crude oil by natural processes [6].

Considering the lack of ability of any bacterial strain for complete catabolism and biodegradation of crude oil, finding appropriate strains is necessary. Even microorganisms which are consumers of non-hydrocarbonate components occasionally play important roles in removal of crude oil from the environment [7].

Many methods have been used to remove oil spills from water including physical removal of crude oil, chemical remediation of the spills using dispersants and so-called “sinking agents,” and, in some cases, intentionally burning floating petroleum slicks. Using chemical dispersants as an oil spill countermeasure is the most frequently employed clean-up method because such liquids can be readily applied to large oil spills, and also this method is generally more cost effective than physical remediation methods [3, 4].

Furthermore, the exclusive property of these dispersants to make oil spills dispersed into water will enhance the biodegradability of crude oil due to the increased exposed surface of the spills to such agents [8].

Chemical dispersion of an oil slick increases the petroleum toxicity, When the meteorological conditions induce the dispersion of the oil slick such as wave, the application of dispersant does not rise the petroleum toxicity [5]. The significant differences between chemically dispersed oil and water-soluble fraction of oil highlight the environmental risk to disperse an oil slick. The lack of significance between chemically and mechanically dispersed oil suggests that dispersant application is no more toxic than the natural dispersion of the oil slick [9].

Dispersants contain surfactants, which are surface-active agents with molecules composed of groups of opposing polarity and solubility; that is, surfactants usually have both an oil-soluble hydrocarbon chain and a water-soluble group. The synthetic surfactants can be anionic, cationic, nonionic, or amphoteric; however, only anionic or nonionic surfactants are utilized as crude oil dispersants. Surfactant mixtures often include other chemical agents, such as solvents, which enhance the dispersing capability of the surfactant [10].

Chemical surfactants are amphiphilic compounds which can reduce surface and interfacial tensions by accumulating at the interface of immiscible fluids and increase the solubility and mobility of hydrophobic or insoluble organic compounds [11–13].

Chemical surfactants can increase the pseudosolubility of petroleum components in water [14, 15].

They are effective in reducing the interfacial tensions of oil and water in situ, and they can also reduce the oil viscosity and remove water from the oil prior to processing [16, 17].

Nevertheless, not all surfactant compositions are efficient in dispersing spilled oil products, and many of the effective ones have the drawbacks of being toxic and/or not biodegradable [4]. Biodegradability of such components is absolutely crucial; otherwise, they get accumulated in the environment and make the secondary cause of water contamination. Modern-day dispersants are much less toxic to sea water than those used in the past. However, concern still exists on their possible toxic effects, on fresh water organisms, especially if dispersants are used near shore waters [18].

This study was conducted to determine the most effective and biodegradable dispersant of spilled oil in water surrounding two Persian Gulf provinces.

## 2. Methods and Materials

We selected two provinces in Persian Gulf because of their location in one of the biggest offshore oil drilling rig and high traffic density of oil vessels in the sea lanes of Persian Gulf; Siri and Bahregan provinces were studied because of their numerous shorelines contaminated by oil spills. We compared the effects of the dispersants produced by Iranian Offshore Oil Company, that is, Pars 1 Pars 2 in comparison with Gamlen, which is actually used in the Persian Gulf water. Biodegradability of Gamlen and its effects on dispersing of oil had already been studied [11, 19].

The culture used in the investigation was originally isolated from 16 sampling stations located at different longitude and latitude in the Siri and Bahregan province offshore (Table 4).

Microorganisms were isolated through common microbiological experiments under the sterile condition. They were cultured in the presence of Pars 1, Pars 2, and Gamlen separately and also the combination of each with oil at a dispersant-to-oil ratio of 1/20 [10, 20]. Dispersants were the only source of carbon in the culture. The ability of bacteria and fungi to grow at the sides of wells was assayed using the Well method and their growth rate was measured through optical density reading.

In this regard, isolated microorganisms, which were able to grow at the side of wells, were individually cultured overnight. 100 mL of manual culture media ((concentration in 1/mg): KH_2_PO_4_ (170), K2HPO4 (435), Na_2_HPO_4_ 7H_2_O (668), NH_4_Cl (850), MgSO_4_·7H_2_O (22.5), CaCl_2_·2H_2_O (27.5), and FeCl_3_ (0.25)) and 2 mL of fresh overnight culture of each microorganisms were poured into six 500 mL flasks. Dispersants as an exclusive source of carbon were added to each flask as follows: flask 1: Pars 1, flask 2: Pars 2, flask 3: Gamlen, flask 4: Pars 1 and oil, flask 5: Pars 2 and oil, flask 6: Gamlen and oil.

 It should be noted that the series of flasks containing both dispersant and oil was set up by preparing 1 : 20 dilution of the dispersant/crude oil [10]. Six control flasks contained sterile media, dispersants, and/or crude oil. All flasks were incubated at 30°C shaking at 120 rpm for 28 days. In different time intervals (24, 48, and 72 hours and 1, 2, 3, and 4 weeks), the optical density of each sample was measured using spectrophotometer at wave length of 600 nm. Oil components and dispersant were isolated from each sample prior to oxygen demand (OD) reading using Hexane Normal [21].

In order to identify the biodegradation of above-mentioned dispersants and their mixture with oil in the sampling stations, the BOD and the COD of each sample were measured in the presence of total microorganisms [22, 23]. The procedure was as follows: 1 mL of water obtained from each station was inoculated into marine broth in 8 screwed cap tubes and one tube was kept as control. Marine broth which is an enriched bacterial medium was used for enhanced growth of all type of microorganisms. After incubating the tubes at 37°C for the period of 48–72 hours, all culture media of 8 tubes containing the grown microorganisms were put together in nutrient broth media and were cultured over night. Therefore, a culture media containing all microorganisms obtained from the sampling stations was prepared. Then, 1 mL of bacterial suspension obtained from total microorganisms' culture was inoculated to each flask containing 100 mL of manual media. Furthermore, the component was singly added to the series of flasks so that each flask contained only one type of dispersant or its mixture with oil as follows: 1 mL crude oil, 1 mL Pars 1, 1 mL Gamlen, 1 mL Pars 2, 1 mL mixture of crude oil and Pars 1, 1 mL mixture of crude oil and Pars 2, 1 mL mixture of crude oil and Gamlen, and their BOD and COD were measured after 5 days using the standard method [24].

The standard microbiological procedures were used to identify the species of the bacterium and fungus microorganisms.

### 2.1. Statistical Analysis

The obtained data was analyzed by the Statistical Package for Social Sciences software version 15.0 (SPSS Inc, Chicago, IL, USA) using analysis of variance (ANOVA) and post hoc Tukey statistical tests.

## 3. Results

Based on standard microbiological procedures, 4 genera of bacteria and 3 genera of fungus in Siri province and 4 genera of bacterium and 2 genera of fungus in Bahregan province were identified.

### 3.1. Results Obtained from the Well Method

**Figure 1:**
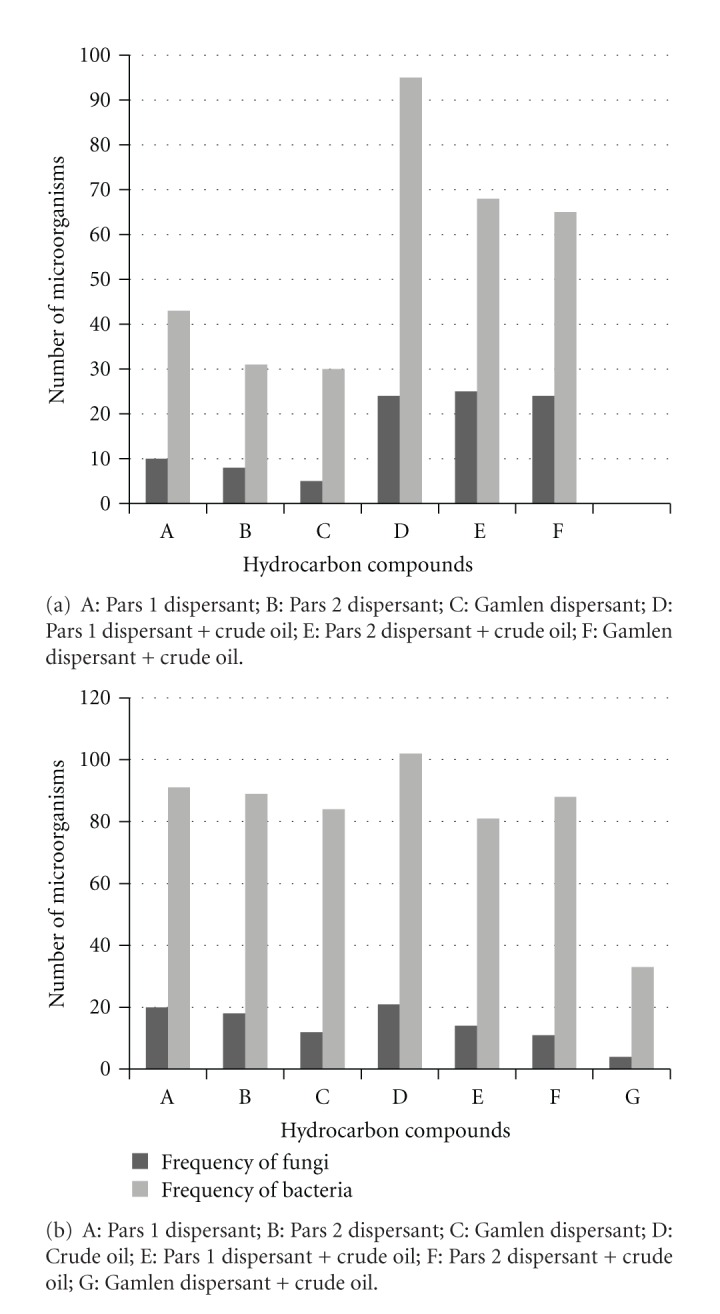
Abundance of microorganism's growth at the side of wells containing organic components: (a) Siri province; (b) Bahregan province.

The ability of bacteria and fungi to grow in the presence of dispersants individually or mixture of each with crude oil is presented in Table 1 and Figure 1.

The highest number of bacteria and fungi in Siri Province were those cultured in the presence of Pars 1, Pars 2 or separate mixture of each with crude oil, whereas in Bahregan Province, the bacteria around Pars 1 and Pars 2, had better growth rate compared with Gamlen.

### 3.2. Biomass Analysis of Bacterial Culture Using the Optical Density Reading

In Siri province, the microorganisms isolated from the sampling stations, which had the ability to grow at the side of wells in the Well method, were cultured 28 days for the purpose of biomass analysis. The bacteria and fungi were grown for 28 days, and their optical density was measured in different time intervals. The OD reading showed that the highest growth occurred in the presence of either Pars 1 or Pars 2 or their separate mixture with crude oil.

The results of ANOVA and post hoc Turkey statistical tests are shown in Table 2.

As shown in Table 2, in Siri province significant differences (*P* = 0.006) were documented in the effects of isolated microorganisms (4 bacteria and three fungi) on different dispersants (Pars 1, Pars 2, and the combination of Gamlen dispersant with crude petroleum) after 24 hours.

The comparison of the mean growth rate using hoc Tukey test exhibited that there is meaningful differences between Pars 1 and Gamlen dispersants (*P* ≤ 0.046) and also between Gamlen and combination of crude oil with Pars 1 (*P* ≤ 0.005). In other words, the effect of microorganisms on Pars 1 dispersant is more than that on Gamlen dispersant.

 This finding is in agreement with the result obtained from the Well method experiment.

The results of ANOVA test on day 28 did not show significant difference in effects of microorganisms among each dispersant separately or their combination with crude oil.

The results of ANOVA test on day 28 (*P* ≤ 0.755) showed that there is no meaningful differences in effects of microorganisms among each dispersant separately or their combination with crude oil.

**Figure 2:**
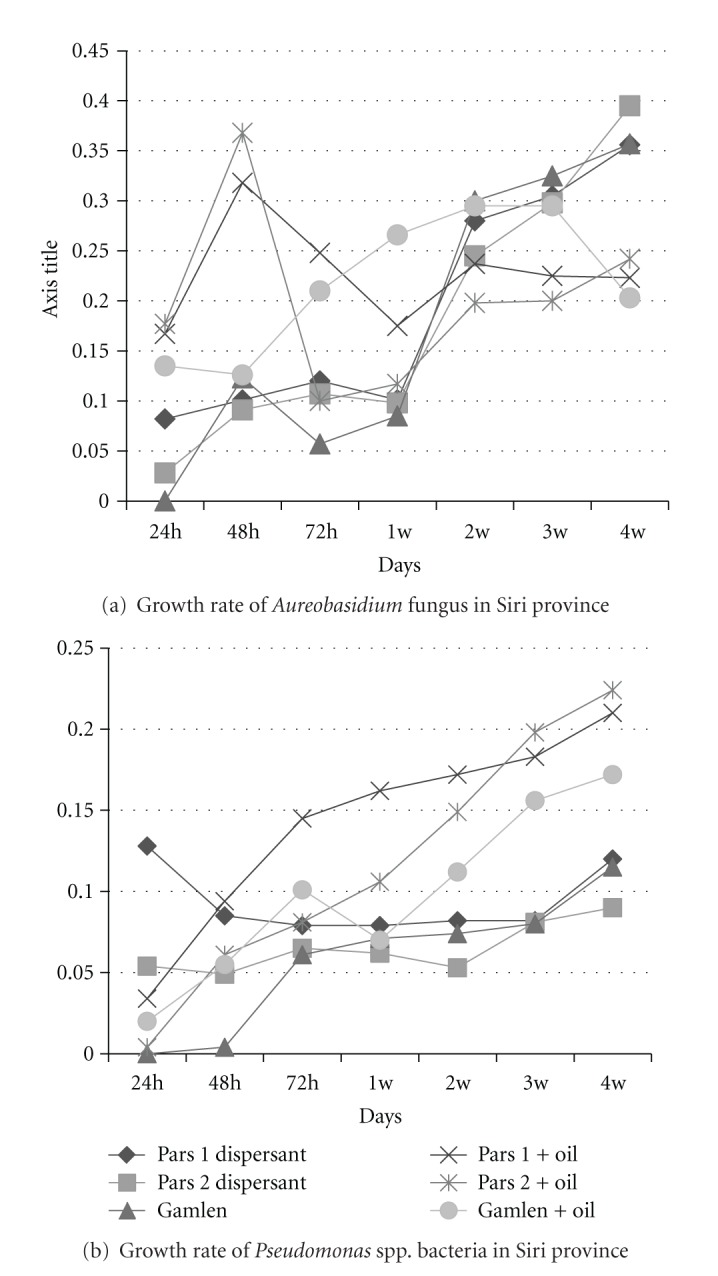
Growth rate of fungus and bacteria in Siri province.

The optical density of one sample of bacterial and fungal cultures with the highest absorbance during 28 days in Siri province is depicted in Figures 2(a) and 2(b).

As shown in Figure 2(a), on the day 28, the highest growth rate of *Aureobasidium* spp. fungus in Siri province was observed in the presence of Pars 2 dispersant and the combination of Pars 2 dispersant with crude oil. The growth pick occurred in the first 24 hours in the presence of combination of Pars 2 dispersant with crude oil. However, the growth rate of microorganisms in the presence of Pars 1 dispersant was also noticeable.

As shown on Figure 2(b), on day 28, the highest growth rate of *Pseudomonas* spp. bacteria in Siri province was high when they were grown in the presence of combination Pars 2 dispersant with crude oil and. On day 14, high growth rate of the microorganism was observed in the presence of combination of Pars 1 dispersant with crude oil.

**Figure 3:**
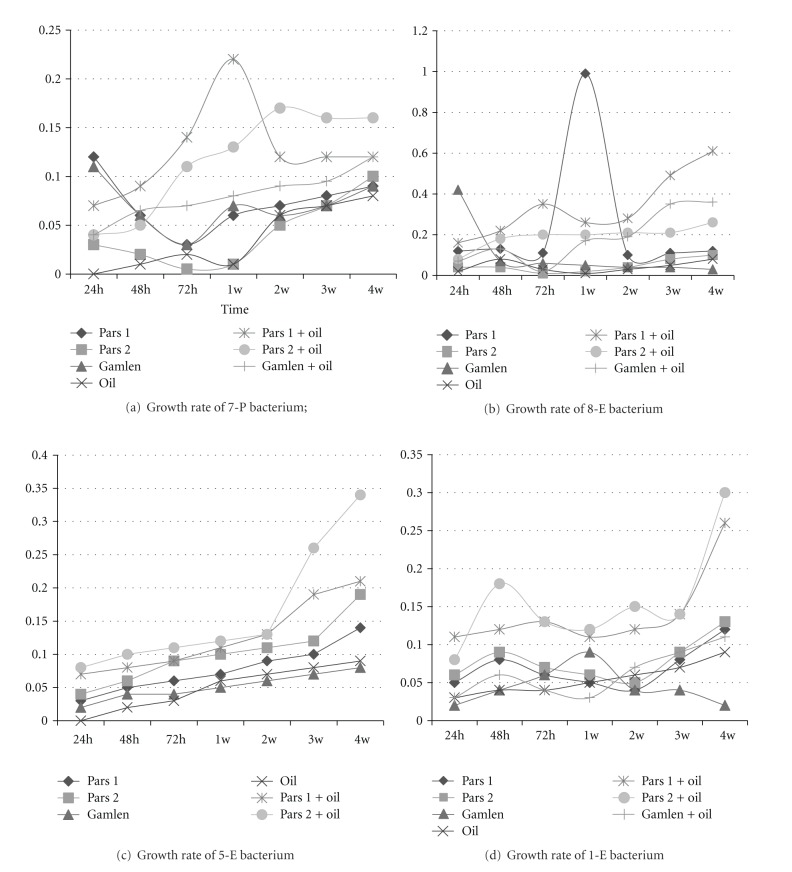
Growth rate of bacteria in the Bahregan province.

In Bahregan, province, the absorbance rate of samples during 24, 48, 72 hours, and 1–4 weeks was measured with spectrograph in 600 nm wave length. Moreover, according to Figures 3(a), 3(b), 3(c), and 3(d) that show the growth rate in 4 bacteria of Bahregan, the maximum rate was when they were grown in the presence of Pars 1 and Pars 2 dispersants.

### 3.3. Results of BOD-COD Tests

The results from BOD and COD analyses are shown in Table 3; the BOD and COD levels in culture having only Siri province crude oil were very low, but, when treated by dispersants, the levels increased. However, as the results show among different combinations of crude oil and dispersants in Siri province, the mixture of crude oil and Pars 2 had the highest BOD and COD followed by the mixture of crude oil and Pars 1. Moreover, among the three different dispersants under study, Pars 1 had the highest BOD and COD. However, In Bahregan Province the maximum level of BOD was devoted to Pars 2, Pars 1 and Gamlen, respectively. The combination of crude oil and dispersants had similar effect with Pars 1 and Pars 2, and it was more than the combination of Gamlen with crude oil.

The highest COD was related to Pars 2, Gamlen, and Pars 1, respectively. Moreover, the highest amount of COD is related to combination of Pars 2 with oil.

The overall results show that Pars 1 and Pars 2 dispersants produced by the Iranian Offshore Oil Company were more effective than Gamlen dispersant in Siri and Bahregan provinces.

## 4. Discussion

In this study, we compared the effectiveness of three dispersants on removal of oil spills; since the highest numbers of microorganisms grown around wells containing Pars 1, Pars 2 and also their separate mixtures with crude oil, our findings suggest that both Pars 1 and Pars 2 dispersants are more effective in biodegradation of crude oil. Furthermore, they have more biodegradability and bioadaptability properties compared with the Gamlen dispersant. We found that by using the Well method experiment, the highest number of isolated bacteria and fungi was able to grow at the side of wells containing Pars 1 dispersant. The growth index represents the mass of microorganisms at the side of wells containing dispersants which indicates the ability of microorganisms to use crude oil and dispersant components [20]. Therefore, microorganisms grown around the wells are considered as petroleum-degrading microorganisms. In this agreement, the highest optical density was related to the bacterial culture containing above-mentioned dispersants as the exclusive carbon source.

The result of statistical calculations on isolated bacteria (4 genera) and fungi (3 genera) in Siri province showed that in the first 24 hours, noticeable growth has occurred in the presence of Pars 1 dispersant as well as its combination with crude oil showing significant difference compared to the growth of the same organisms in the presence of the other dispersants or their combination with Siri province crude oil. In Bahregan province, in all conditions the maximum growth rate was found in the presence of Pars 1 and Pars 2 in the environment. In 4 bacteria of Bahregan province, combination of crude oil with 2 bacteria of 5-E and 1-E which are related to *Flavobacterium* spp. and *Enterobacter* spp., respectively, showed higher degradability and growth rate. However, in 2 other bacteria which are related to Pseudomonas spp and Bacillus genus (7-P and 8-E), the highest growth rate is when Pars 1 and Pars 2 are the only sources of carbon in the environment. In 7-P bacterium the highest growth rate was observed in the presence of Pars 1 dispersant after one week. 8-E bacterium had the highest growth in the first week in the presence of Pars 1 dispersant and in 4th the week with presence of combination of Pars 1 and crude oil. Moreover, 5-C and 1-E showed the highest growth rate after four weeks in the presence of Pars 2 dispersant.

These findings confirm that Pars 1 dispersant has more adaptability to both province ecosystems, and also they have more ability in biodegradation of crude oil compared with the other two dispersants. There was no significant differences in the effect of microorganisms on each dispersant and also on their combination with crude oil after 28 days. However, since the entered material to the ecosystem needs to be degraded fast, Pars 1 dispersant which shows more degradability in the first 24 hours comparing other dispersants, is more adaptable to the environment.

In Siri province, The growth diagrams of *Pseudomonas* spp. bacteria in 4 weeks showed the highest growth rate of bacteria to the great extent in the presence of Pars 1 dispersant and slightly lower extent in the presence of Pars 2 dispersant. The growth peak was seen on day 14 in the presence of combination of Pars 1 dispersant and crude oil. The growth curve of *Aureobasidium* spp. fungus shows that on day 28, the highest optical density is observed when the microorganism is cultured in the presence of Pars 2 and its mixture with crude oil; also the growth peak occurred in the presence of the aforementioned mixture in the first 24 hours. However, the microorganisms had also enhanced growth in the presence of Pars 1 dispersant.

 The optical density of microorganism cultures containing dispersants is indicating the usage of these components as the sole carbon source which leads to microorganism growth and catabolism of the carbohydrate [11, 25]. Therefore, the result of the OD reading and the Well method experiments show that Pars 1 and Pars 2 are more effective in biodegradation of crude oil of both provinces, also they are more biodegradable than Gamlen dispersant. Overall, these findings suggest that Pars 1 and Pars 2 are more bio-adaptable for both province offshores.

 In agreement with results obtained from optical density reading and the Well method experiment, microorganism culture containing Pars 1 showed the highest level of BOD and COD. BOD is an indicator of biodegradation of organic components in water. BOD is measured by the amount of required oxygen for bacteria to metabolize organic components. The flasks are kept at 20°C for 5 days and the amount of dissolved oxygen is determined by chemical procedures. The BOD test identifies the approximate amount of required oxygen for biological oxidation of contaminated water, surplus water, and sewages. This is the only experiments determining the amount of required oxygen for bacteria in order to catabolize the organic components. Therefore, the higher BOD shows the increased amount of consumed oxygen which is consequently indicating the enhanced bacterial activity [24].

The COD value indicates the amount of oxygen needed to chemically oxidize organic compounds present in wastewater and adjacent to oxidizing material. In fact, chemical oxygen demand determines the amount of organic compound present in the sample which has the ability to be oxidized by a strong chemical oxidizing agent [24]. 

BOD and COD tests are well-known methods for assessment of biodegradability of organic materials such as surfactants; for instance, in a study in 1976, the percentage of biodegradation of 123 organic compounds was assayed [23]. 

Previous studies showed that none of the bacterial species are able to catabolize all the components of crude oil, and its complete biodegradation depends on the presence of various bacterial species and microorganisms. Even microorganisms which are consumers of non-hydrocarbonate compounds can play important role in biodegradation of crude oil [7]. Thus, in the present study, we measured the BOD and the COD of all microorganism cultures, which were originally isolated from sampling stations in the Persian Gulf in the presence of three dispersants and their mixtures with crude oil and also crude oil alone.

 In this study, the highest BOD and COD in Siri province were related to culture containing Pars 1 in comparison with the two other dispersants studied.

 Likewise, BOD-COD test performed on different combinations of dispersants and crude oil showed highest score for the combination of Pars 1 and crude oil and secondly to the mixture of Pars 2 and crude oil. Moreover, in Bahregan province the maximum BOD and COD devoted to Pars 2 dispersant. These findings suggest that microorganisms with the presence of these compounds require the highest amount of oxygen for their activities including the biodegradation of such components. Furthermore, in oxidation and degradation reactions, in presence of oxidizing agents, the highest amount of chemical oxygen is demanded; therefore; Pars 1, mixture of Pars 2 and crude oil, and also mixture of Pars 1 and crude oil hold more degradability properties compared to the mixture of Gamlen and crude oil. In fact, Pars 1 and Pars 2 increased biodegradability of crude oil more than that of Gamlen dispersant. Since Pars 1 showed more degradability compared with the other two dispersants, the bioadaptability of this dispersant is high enough, so that it does not get accumulated in the region and does not make the environment contaminated.

## 5. Conclusion

This study revealed that Pars 1 and Pars 2 dispersants are more biodegradable than Gamlen and have more effectiveness in biodegradation of crude oil. These findings suggest that, in each region, the most suitable compound for removing oil spill from offshores with least secondary contamination should be investigated.

## Figures and Tables

**Table 1:** Frequency of microorganisms grown at the side of wells in the Well method.

Variables	Frequency of fungi	Frequency of bacteria
Siri province		
Pars 1 dispersant	10	43
Pars 2 dispersant	8	31
Gamlen dispersant	5	30
Pars 1 + crude oil	24	95
Pars 2 + crude oil	25	68
Gamlen + Petroleum	24	65

Bahregan province		
Pars 1 dispersant	20	91
Pars 2 dispersant	18	89
Gamlen dispersant	12	84
Pars 1 + crude oil	14	81
Pars 2 + crude oil	11	88
Gamlen + Petroleum	4	33

**Table 2:** The effect of isolated microorganisms on Hydrocarbonate compounds in 24 hours.

Compounds of hydrocarbon	Mean	SD
Pars 1 dispersant	0.126	0.082
Pars 2 dispersant	0.055	0.049
Gamlen dispersant	0.011	0.004
Crude oil	0.072	0.046
Pars 1 dispersant + petroleum	0.156	0.098
Pars 2 dispersant + petroleum	0.104	0.087
Gamlen dispersant + petroleum	0.115	0.067

Coefficient *F* = 3.53, *P* = 0.006, SD: standard deviation.

**Table 3:** The measurements of BOD and COD (mg/mL).

	Crude oil + Gamlen	Crude oil + Pars 2	Crude oil + Pars 1	Gamlen	Pars 2	Pars 1	Petroleum
Siri province							
BOD_5_ test (mg/mL)	1185	4170	1540	5392	500	9200	65
COD test (mg/mL)	2720	9280	3120	11200	960	16800	128

Bahregan province							
BOD_5_ test (mg/mL)	900	1120	1120	5995	7100	6000	110
COD test (mg/mL)	1840	2880	1840	12480	15200	10560	240

BOD: biological oxygen demand; COD: chemical oxygen demand.

**Table 4:** Longitude and latitude coordinates of the sampling stations.

Station number	Longitude	Latitude
Siri province		
(1)	54°33/410 E	25°54/750 N
(2)	54°34/027 E	25°54/921 N
(3)	54°34/466 E	25°55/141 N
(4)	54°30/133 E	25°50/545 N
(5)	54°27/344 E	25°48/738 N
(6)	54°24/569 E	25°46/414 N
(7)	54°21/100 E	25°44/544 N
(8)	54°18/005 E	25°42/156 N

Bahregan province		
(1)	49°29/100 E	32°29/335 N
(2)	49°30/027 E	32°29/251 N
(3)	49°30/445 E	32°33/251 N
(4)	49°29/441 E	32°30/334 N
(5)	49°27/302 E	32°28/336 N
(6)	49°21/244 E	32°27/416 N
(7)	49°19/340 E	32°29/358 N
(8)	49°19/004 E	32°27/779 N
